# Growth inhibition of arable weeds by cerato‐platanin, a plant immune defense activator of fungal origin

**DOI:** 10.1002/ps.8963

**Published:** 2025-06-24

**Authors:** Laura Scarabel, Andrea Milani, Sihem Fodil, Giorgio Carollo, Simone Luti, Ivan Baccelli

**Affiliations:** ^1^ Institute for Sustainable Plant Protection, National Research Council of Italy Legnaro Italy; ^2^ Institute for Sustainable Plant Protection, National Research Council of Italy Sesto Fiorentino Italy; ^3^ Department of Experimental and Clinical Biomedical Sciences University of Florence Florence Italy

**Keywords:** mode of action, bioherbicide, PAMP‐triggered immunity, ryegrass, sustainable agriculture, fungal protein

## Abstract

**BACKGROUND:**

The herbicide sector needs new modes of action and new ecofriendly molecules as active ingredients. In this study, we investigated the stimulation of the plant immune system as a strategy to reduce weed growth, a mechanism not exploited by any commercial herbicide. Plants possess an innate immune system able to detect pathogens' molecules such as cerato‐platanin (CP), a fungal protein elicitor produced by *Ceratocystis platani*. As sensitivity of arable weeds to microbial elicitors is mostly unknown, the ability of CP to reduce germination and early seedling growth was examined in some Poaceae and Amaranthaceae by using wheat as an off‐target species.

**RESULTS:**

CP‐treated seeds from *Lolium multiflorum*, *Digitaria sanguinalis* and *Amaranthus hybridus* resulted in stunted seedling growth, demonstrating sensitivity to the protein. In contrast, *A. palmeri*, *A. tuberculatus* and *Avena fatua* were unaffected by the treatment. A more detailed characterization of the sensitivity of *L. multiflorum* showed that CP‐specific growth inhibition occurred at concentrations ≥75 μM. Western‐blot analysis showed absorption of CP by *L. multiflorum* seedlings, while RT‐qPCR analyses revealed the overexpression of defense genes, such as the pathogenesis‐related (PR) gene *chitinase 1* and the ethylene‐biosynthesis gene *ACO1*. CP was also effective on *Lolium* spp. populations resistant to the acetyl‐coenzyme A carboxylase (ACCase) inhibitor pinoxaden, while it did not adversely affect germination of *Triticum aestivum*.

**CONCLUSION:**

Besides the classic use as resistance inducers against pests and diseases, we show that plant immune defense activators from microbes may act as selective herbicides with a mode of action so far unexploited. © 2025 The Author(s). *Pest Management Science* published by John Wiley & Sons Ltd on behalf of Society of Chemical Industry.

## Graphical abstract

Plants possess an innate immune system able to detect pathogens' molecules such as cerato‐platanin (CP), a protein produced by the fungus *Ceratocystis platani*. This study shows that the application of this protein elicitor to weeds can be exploited as a novel herbicide mode of action.

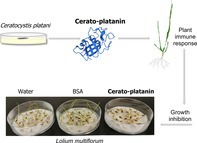

## 1 INTRODUCTION

Discovery and commercialization of herbicides with totally novel mechanisms of action has stalled over the last 40 years.^1^, ^2^, ^3^ Most new active ingredients target the same molecular pathways as older herbicides.^4^ On the other hand, the number of herbicide resistant weeds is constantly rising and most existing modes of action have been already affected.^5^, ^6^ Resistance problems are exacerbated in countries where, to reduce risk and impact of pesticides, there is pressure to withdraw molecules with problematic eco‐toxicological profiles, or where highly simplified agricultural practices are applied, such as the cultivation of GMO crops. In both cases, there is an over‐reliance on a few chemicals, *de facto* favoring the rise of resistance.^7^, ^8^ In this scenario, the scientific community constantly highlights the need to discover new herbicide modes of action.^2^, ^3^, ^9^, ^10^, ^11^

Living organisms or natural molecules derived from them may represent a powerful source of pesticides with novel modes of action^12^ and a sustainable alternative for integrated weed management strategies.^13^ Some molecules can affect more than one molecular target, thus limiting the risk of evolving resistance.^14^ Nevertheless, only a dozen bioherbicides have been marketed since their first introduction in 1980.^13^, ^15^ The majority are made of selected fungal or bacterial strains, whereas the molecules marketed to date are only pelargonic (nonanoic) acid and thaxtomin A, of plant or bacterial origin, respectively.^13^, ^15^

On the other hand, synthetic herbicides are represented by a very broad array of molecules that are distributed among 24 mode‐of‐action groups. Synthetic herbicides hit key plant processes such as photosynthesis, the biosynthesis/assembly of sugars, amino acids, lipids and nucleic acids, the biosynthesis/regulation of proteins, the auxin transport and activity (HRAC global herbicide MOA classification 2024, https://www.hracglobal.com/).

To date, no herbicide has as its main target the immune system of plants. More precisely, while disease induction with weed pathogenic microbes is a long‐standing concept (e.g., mycoherbicides),^15^ no herbicidal mechanism described to date targets the plant immune response by eliciting (activating) plant immune responses in the absence of any microbial presence. Advancements in plant immunity research have revealed that plants, lacking mobile immune cells, rely on innate immune receptors to detect pathogen‐ or damage‐associated molecular patterns (PAMPs or DAMPs).^16^, ^17^ This recognition triggers defense responses at the molecular and cytological levels^17^, ^18^, ^19^ that may even lead to leaf necrosis^20^ or abscission.^21^ Immune activation incurs metabolic costs and growth‐defense trade‐offs for the plant^22^, ^23^ that limit the practical use of elicitors to protect crops from pathogens and insects.^24^, ^25^ Interestingly, the exploitation of this trade‐off for weed control has remained so far unexplored.

The activation of the first line of immune defense, the so‐called ‘pattern‐triggered immunity’ (PTI),^17^ has been demonstrated in many studies to reduce seedling growth after PAMP or DAMP treatment.^19^, ^26^, ^27^, ^28^, ^29^ With our work, we aimed to investigate immune defense stimulation, specifically PTI, as a strategy to hamper weed growth. To this purpose, we used a fungal PAMP protein named cerato‐platanin (CP), extensively characterized by our research group over the last decades, known to activate immune defense responses in different plant species, such as *Arabidopsis thaliana* and the tree *Platanus acerifolia*.^30^, ^31^, ^32^ CP, a natural product of the fungus *Ceratocystis platani*,^33^ belongs to the homonymous family ‘Cerato‐platanin family’ (InterPro entry IPR010829), which includes proteins exclusively present in the fungal kingdom that are able to stimulate PTI in plants.^19^, ^34^, ^35^

In this work, we first investigated the seedling growth inhibition ability of CP on *Lolium multiflorum* (Italian ryegrass), one of the most problematic weeds infesting cereal fields, vineyards and orchards that has evolved resistance, to date, to eight different mechanisms of herbicidal action.^36^ Subsequently, we tested *Lolium* spp. populations that are resistant to an acetyl‐coenzyme A carboxylase (ACCase) inhibitor, as well as other important arable weeds belonging to the Poaceae and Amaranthaceae families, whose evolution of herbicide‐resistant populations has been reported. We finally investigated the absorption of CP in the seedlings and the expression of pathogen defense genes.

## 2 MATERIALS AND METHODS

### 2.1 Plant material

The seeds used in this study were present in the collection of the Institute for Sustainable Plant Protection of the National Research Council of Italy (IPSP‐CNR, Legnaro, PD). Seeds from each weed species had been collected from different sites from at least 30 mature plants, cleaned, dried at 20 °C for 1 week and stored in paper bags at 4 °C until the beginning of the experiments. The origin of the seeds used in this work is reported in Table 1.

**Table 1:** Origin of the seeds used in this work

Plant species	Population code	Origin
*Amaranthus hybridus*	17‐53‐L	Natural park, Rome (Italy)
*Amaranthus palmeri*	20–179	Provided by Prof. T. A. Gaines (Colorado State University, USA)
*Amaranthus tuberculatus*	17–65	Sandy mud flat, Portalbera, PV (Italy)
*Avena fatua*	6‐95‐L	Wheat field, Torre Maggiore, FG (Italy)
*Digitaria sanguinalis*	18–8	‘L. Toniolo’ experimental farm, Legnaro, PD (Italy)
*Lolium multiflorum*	204‐L	Wheat field, Civitella Paganino, GR (Italy)
*Lolium* spp.	19–670	Wheat field, Imola, BO (Italy)
*Lolium* spp.	IT609	Wheat field, Alessandria, AL (Italy). Resistance to both ALS (mesosulfuron‐methyl and iodosulfuron methyl‐sodium) and ACCase (pinoxaden and clodinafop‐propargyl) inhibitors. Population characterized by both target‐site and metabolism‐based resistance.^37^
*Lolium* spp.	23–735	Wheat field, Torre San Patrizio, FM (Italy)
*Lolium* spp.	23–741	Wheat field, Perugia, PG (Italy)
*Triticum aestivum*		Purchased at Soc. Cooperativa Agricola Piovese, Arzergrande, PD, (Italy)

### 2.2 Cerato‐platanin (CP) production and purification

The water‐soluble protein cerato‐platanin (CP), originally purified from the ascomycete fungus *C. platani*,^33^ was produced by heterologous expression in the yeast *Pichia pastoris* according to the method described by Carresi *et al*.^38^ The expression of CP in *P. pastoris* allows obtaining higher yields, a correct folding, while maintaining the original eliciting and chitin‐binding properties of the protein, as demonstrated in previous studies.^31^, ^39^, ^40^ CP was purified by Reverse Phase‐High Performance Liquid Chromatography (RP‐HPLC), quantified by the bicinchoninic acid (BCA) method and checked by SDS‐PAGE analysis.^38^

### 2.3 Seed treatment and seedling growth measurements

Before the treatments with CP, the seeds were surface sterilized in 1% (v/v) sodium hypochlorite solution for 5 min., washed twice with sterile water and left to dry for a few minutes on a paper sheet.

The activity of CP was first assessed in *L. multiflorum* 204‐L. Thirty seeds were placed on a disc of sterile filter paper in a 60 × 15 mm Petri dish working under a laminar flow hood. Treatments were performed by dispensing on the paper 500 μL of 150 μM CP. This concentration was selected based on previous studies showing that 150 μM CP can induce defense responses and decrease photosynthetic activity in *A. thaliana* leaves.^31^, ^41^ As a control, sterile water and 150 μM Bovine Serum Albumin (BSA, pH 7, purity ≥98%, Sigma‐Aldrich, Merk Life Science S.r.l., Italy) were used. BSA is a protein standard routinely used in biochemistry assays, it is xenobiotic to plants and possesses an isoelectric point similar to CP.^34^ Subsequently, a dose–response assay was performed to identify the minimal inhibitory concentration of CP and assess the specificity of the inhibition. The following serial dilutions were tested on *L. multiflorum*: 37.5, 75, 150, 300 and 600 μM (in water). The specificity of the inhibition was assessed by testing in parallel serial dilutions of BSA (37.5, 75, 150, 300 and 600 μM). Sterile water was used as a control. Each concentration was tested in triplicate by adding 500 μL to three Petri dishes containing 30 seeds each, prepared as previously described. Shoot and primary root length measurements were performed on germinated seeds after 4 days.

The inhibitory effect of CP on different plants was performed as follows. For each species, 30 seeds (or 20 seeds for *Triticum aestivum*) were placed on a disc of sterile filter paper in a 60 × 15 mm Petri dish and treated with 500 μL of 150 μM CP, 150 μM BSA, or water as described above. To facilitate the soaking of *Avena fatua* seeds, the incubation was performed with 1.5 mL of CP, water or BSA. Petri dishes were sealed with parafilm and incubated at 4 °C in the dark for vernalization. After 4 days (21 days for *Digitaria sanguinalis*), the Petri dishes were moved to the growth chamber under the conditions reported in Table 2. Shoot and primary root length measurements were performed only on germinated seeds after 4–6 days, depending on the species (see figure legends).

**Table 2:** Growth conditions

Plant species	Vernalization (days at 4 °C)	Photoperiod (day/night hours)	Growth temperature (day/night °C)	No. of seeds/Petri dish
*A. hybridus*	4	12/12	27/17	30
*A. palmeri*	4	12/12	27/17	30
*A. tuberculatus*	4	12/12	27/17	30
*A. fatua*	4	12/12	25/15	30
*D. sanguinalis*	21	12/12	25/15	30
*Lolium* spp.	4	12/12	25/15	30
*T. aestivum*	4	12/12	25/15	20

To assess the ability of CP to inhibit herbicide resistant populations of *Lolium* spp., the following field populations having different frequencies of resistant plants to the ACCase inhibitor pinoxaden were used: IT609, 17–670, 23–735, 23–741 (Table 1). The susceptible *L. multiflorum* population 204‐L was used as a control. The Petri dish assay was conducted as reported above, with the only difference that the seeds were first vernalized in 500 μL of sterile water, and 500 μL of 150 μM CP were added at the end of the vernalization period. In addition, shoot length measurements were performed after 7 days of growth. To confirm resistance of the above‐mentioned populations to pinoxaden, a Petri dish assay was performed in parallel by adding, at the end of vernalization, 500 μL of an appropriate dilution of Axial® Pronto 60 (Syngenta Italia). Shoot length reduction percentage was calculated per each population, for both CP and pinoxaden, by using the mean value of the water‐treated control.

All the experiments described in this paragraph included at least two biological replicates and were repeated as reported in the figure legends.

### 2.4 SDS‐PAGE and Western blot analyses

To assess whether CP was absorbed during germination/seedling growth, *L. multiflorum* and *T. aestivum* seedlings were analyzed by Western Blot analysis. Seeds were first treated with CP or water, subsequently vernalized as described in the previous paragraph, and grown as summarized in Table 2. After 4 days of growth in three different Petri dishes containing either 500 μL of 150 μM CP (two dishes), or water as a control (one dish), *L. multiflorum* seedlings were collected and subjected to SDS‐PAGE and Western blot analyses. Seedlings from one of the CP‐containing Petri dishes were immediately frozen in liquid nitrogen for protein extraction, while seedlings from the other Petri dish were rinsed twice in sterile water to remove possible external traces of CP and then frozen in liquid nitrogen for protein extraction. The presence of CP within *T. aestivum* seedlings was similarly investigated after 4 days of growth, but three different Petri dishes containing either 500 μL of 150 μM CP, or water as a control, were separately collected and immediately frozen in liquid nitrogen for protein extraction. In addition, a single replicate experiment with *L. multiflorum* was run in parallel and analyzed for comparison.

Frozen tissues were transferred into a mortar containing liquid nitrogen and pulverized with a pestle. The resulting powder was resuspended in phosphate‐buffered saline (PBS) supplemented with a 1× protease inhibitor cocktail (Sigma‐Aldrich, cat. no. P9599). The samples were sonicated on ice with a Fisher Scientific™ Model 120 Sonic Dismembrator (four pulses of 15 s., 75% amplitude, each followed by a cooling period of 30 s.). The sonicated samples were cleared by centrifugation at 15 000 *g* for 10 min. at 4°C. The supernatant was collected and stored at −20 °C for further analysis. Proteins (10 μg per sample) were separated on a 4–20% polyacrylamide gel (Bio‐Rad Laboratories S.r.l., Italy) and either stained with Coomassie Brilliant Blue or transferred onto a PVDF membrane (Bio‐Rad Laboratories). For Western blotting, primary anti‐CP antibodies (1:1000) were used,^42^ followed by horseradish peroxidase‐conjugated anti‐rabbit secondary antibodies (1:5000, Santa Cruz Biotechnology Inc., Germany). Chemiluminescence was achieved by probing the PVDF membrane with electrochemiluminescence substrate (ECL; Bio‐Rad Laboratories, Cat. no. 1705060S). Target protein bands were detected and analyzed by using the Amersham Imager 600 system (GE Healthcare, Italy).

### 2.5 RNA extraction and gene expression analyses

*L. multiflorum* seeds treated with 150 μM CP, 150 μM BSA, or water, were vernalized and incubated as described in Table 2. Total RNA was extracted after 4 days of growth from three biological replicates per treatment, each consisting of a pool of approximately 30 seedlings, by grinding in liquid nitrogen and extracting with RNeasy Plant Mini Kit, buffer RLT (Qiagen, Italy). RNA was quantified in a TECAN Infinite M Plex microplate reader equipped with NanoQuant Plate (Tecan Group Ltd., Switzerland). RNA was first checked for integrity by agarose gel electrophoresis, DNase treated by using Amplification Grade DNase I (Sigma‐Aldrich) and reverse‐transcribed with Maxima First Strand cDNA Synthesis Kit (Thermo Fisher Scientific, Italy). Quantitative RT‐PCRs were performed in a StepOne Plus Real‐Time PCR System (Applied Biosystems, Life Technologies Europe BV, Italy) by using Fast SYBR™ Green Master Mix (Applied Biosystems). The genes analyzed and the corresponding primer sequences are reported in Supporting Information, Table S1. Primers were designed with Primer Express 3.0 (Applied Biosystems). Relative gene expression values were calculated from three biological replicates and two technical replicates by using the 2^−ΔΔCT^ method as described by Livak and Schmittgen,^43^ after melting curve analysis and amplification plot comparisons. The *Eukaryotic translation initiation factor* (*eTIF*) gene was used as the endogenous reference for transcript normalization after verification of its transcriptional stability (Supporting Information, Fig. S1).

### 2.6 Statistical analyses

The effect of the treatments on seedling growth was analyzed by non‐parametric Kruskal–Wallis test followed by Dunn's multiple comparison test (*P* < 0.05). Gene expression data were analyzed by one‐way ANOVA followed by Dunnett's multiple comparisons test (*P* < 0.05). All data were first tested for normality (D'Agostino‐Pearson, Shapiro–Wilk, Anderson‐Darling and Kolmogorov–Smirnov tests) and homogeneity of variance (Bartlett's and Brown‐Forsythe tests). GraphPad Prism 9 (GraphPad Software Inc., CA, USA) was used for graph design and statistical analyses.

## 3 RESULTS

### 3.1 Seed treatment with cerato‐platanin (CP) inhibits *Lolium multiflorum* seedling growth

**Figure 1:**
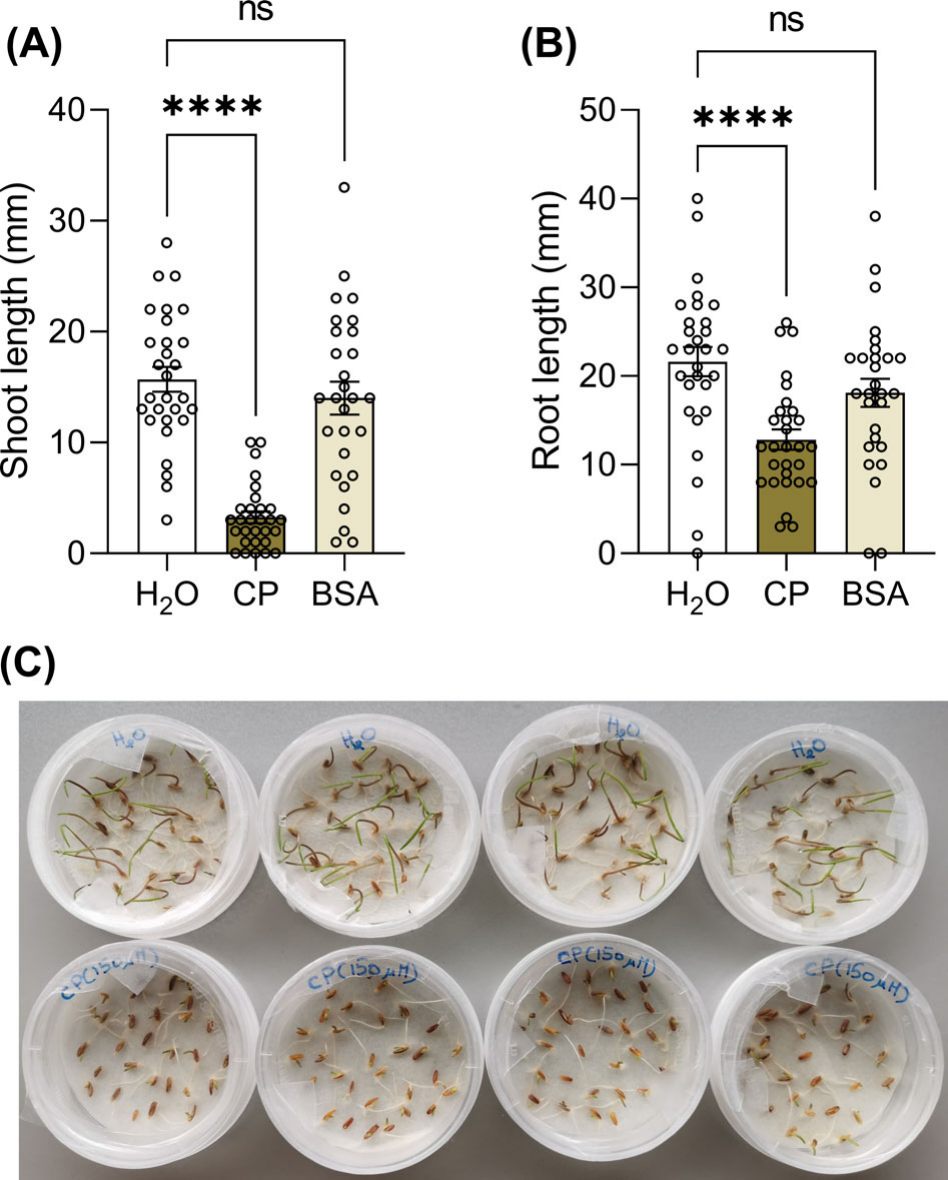
Seedling growth inhibition of *Lolium multiflorum* by cerato‐platanin (CP). *L. multiflorum* 204‐L seeds were vernalized 4 days at 4 °C in the presence of 150 μM CP. Sterile water (H_2_O) and 150 μM bovine serum albumin (BSA) were used as controls. Shoot (A) and primary root (B) length measurements were performed after 4 days of growth. Non‐germinated seeds were excluded from measurements. Statistical significance was analyzed by non‐parametric Kruskal–Wallis test followed by Dunn's multiple comparisons test at *P* < 0.05 (*n* = 28–29). Graphs show average values ± SEM. ****: *P* < 0.0001; ns, not significant. The experiment was repeated three times with similar results. A representative picture (C) of the effects of CP (bottom line) on *L. multiflorum* seedlings is shown (water control, upper line).

*L. multiflorum* was used as the first species to assess the sensitivity of arable weeds to the eliciting activity of CP. As shown in Fig. 1, 150 μM CP, a concentration previously reported to stimulate defense responses in *A. thaliana* leaves,^31^ was able to significantly reduce the shoot and root length of germinated *L. multiflorum* seeds. While the germination rate was unaffected by the presence of CP (96.7% for both H_2_O and CP), the reduction in length caused by CP was approximately 80% for shoots and 40% for roots, respectively, as compared to water control (H_2_O). In contrast, the treatment with the standard protein BSA that we used as a protein control did not significantly affect seedling growth as compared to water (Fig. 1), nor did it considerably affect germination rate (93.3%).

### 3.2 Seedling growth inhibition is dose‐dependent and specific to the protein CP

**Figure 2:**
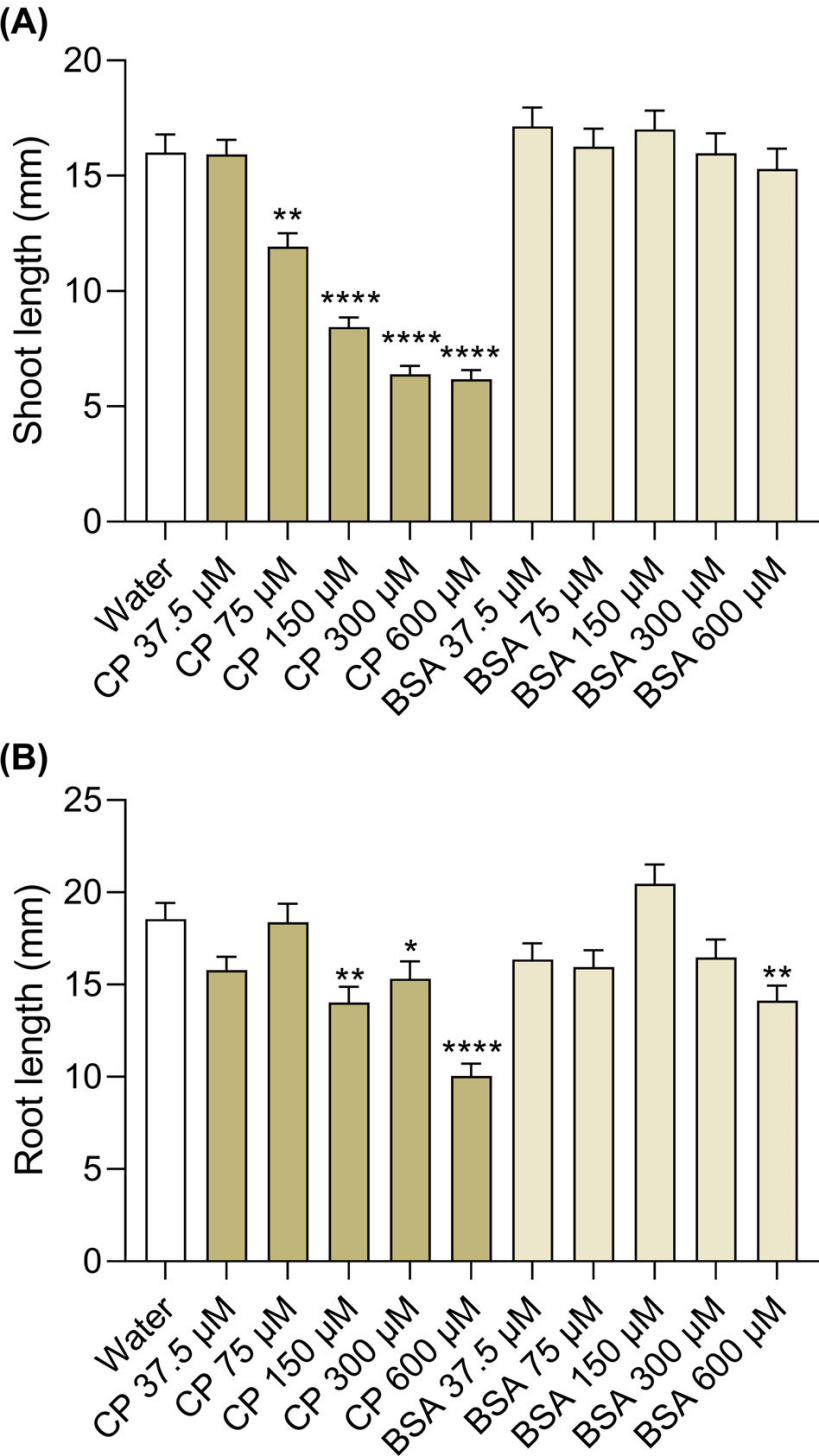
Dose–response effect of cerato‐platanin (CP) on *Lolium multiflorum* growth. *L. multiflorum* 204‐L seeds were vernalized with increasing concentrations of the protein elicitor CP, or the control protein bovine serum albumin (BSA). Shoot (A) and primary root (B) length measurements were performed after 4 days of growth. Non‐germinated seeds were excluded from measurements. Statistical significance was analyzed by non‐parametric Kruskal–Wallis test followed by Dunn's multiple comparisons test (*vs*. water) at *P* < 0.05 (*n* = 84–90). Graphs show average values ± SEM. Significant differences are indicated with *: *P* < 0.05, **: *P* < 0.01, or ****: *P* < 0.0001.

A subsequent seedling‐growth inhibition assay was performed to identify the minimal inhibitory concentration of CP and to assess its specificity. As shown in Fig. 2, while CP was able to significantly reduce shoot length at concentrations ≥75 μM, BSA was not able to inhibit shoot growth with any concentration tested. Concerning root development, a significant inhibition in the presence of CP was observed with a minimum concentration of 150 μM, while a non‐specific effect of the treatment with BSA was observed only with the highest concentration tested (600 μM). The germination rate remained substantially unaffected by the presence of CP at any concentration tested (Supporting Information, Table S2).

### 3.3 CP is absorbed by *L. multiflorum*

**Figure 3:**
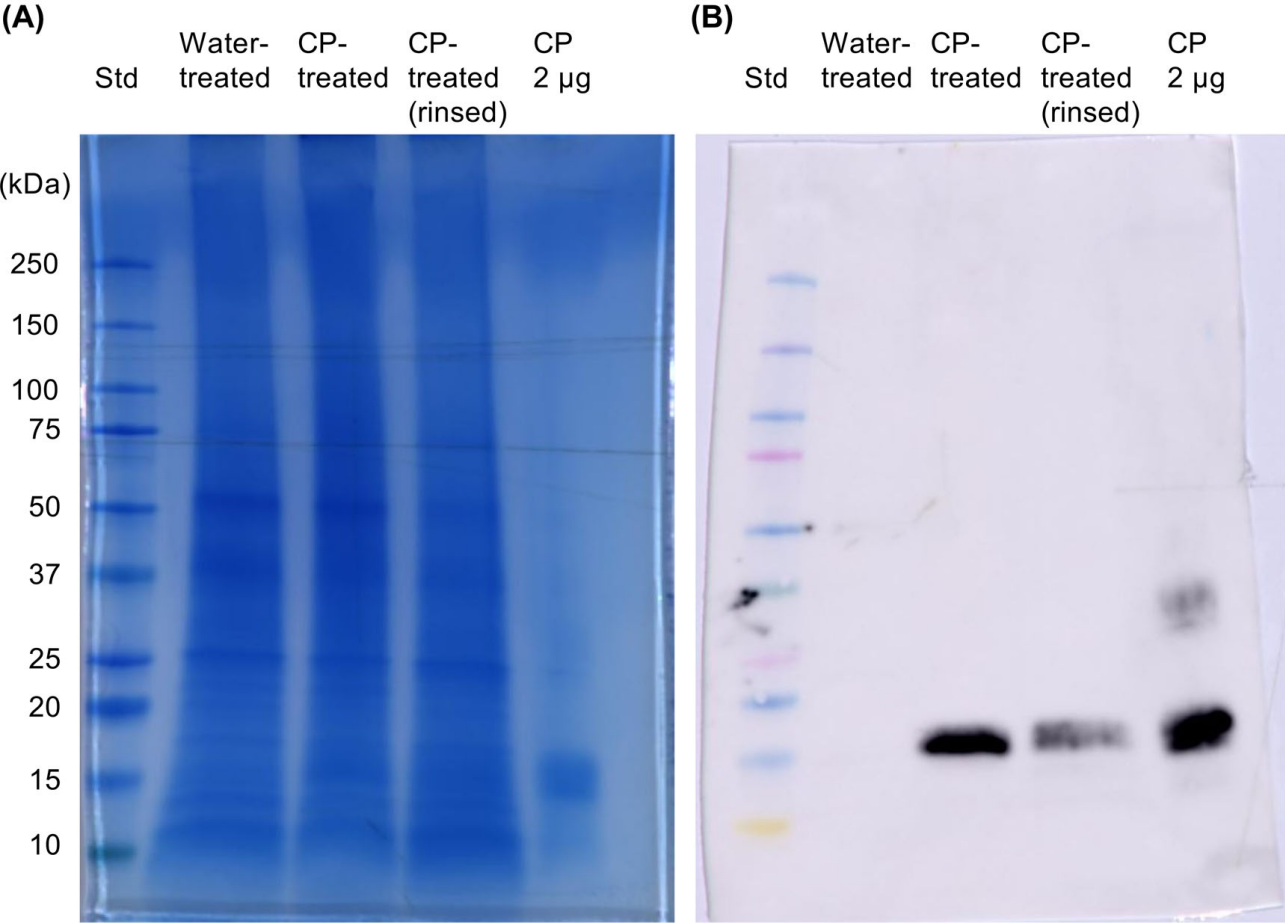
Immunodetection of cerato‐platanin (CP) in *Lolium multiflorum* seedlings. SDS‐PAGE (A) and Western blot anti‐CP (B) analyses of lysates of *L. multiflorum* seedlings grown in water (control) or in the presence of 150 μM CP. Before protein extraction, half of the seedlings germinated with CP were rinsed in water to remove external traces of CP. Pure CP (2 μg) was loaded as a positive control. Std, Precision Plus Protein Kaleidoscope™ Prestained Protein Standards #1610375.

SDS‐PAGE (Fig. 3(A)) and Western blot (Fig. 3(B)) analyses were performed to determine whether CP was absorbed by *L. multiflorum* during germination/early seedling growth. As shown in Fig. 3(B), a distinct band corresponding to CP was observed in the protein extract of both rinsed and non‐rinsed CP‐treated seedlings, while it was absent in water‐treated control. Washing did not completely remove CP from the seedlings, indicating that the protein had been absorbed during germination or seedling growth.

### 3.4 CP alters gene expression in *Lolium multiflorum* seedlings

**Figure 4:**
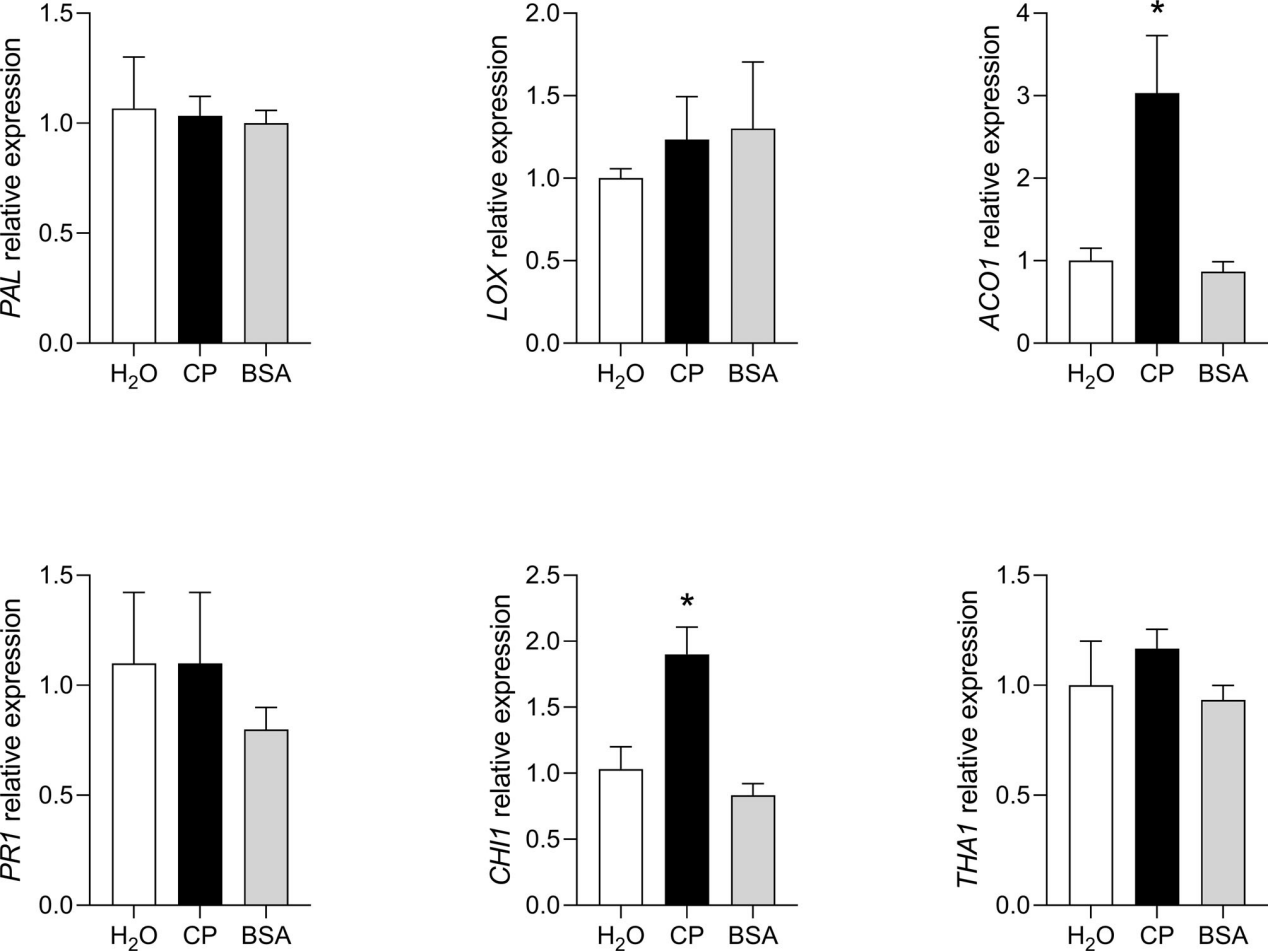
Gene expression analysis in *Lolium multiflorum* seedlings germinated in the presence of cerato‐platanin (CP). CP was applied to seeds before vernalization at the concentration of 150 μM. Water and 150 μM BSA were used as controls. The following genes were analyzed by RT‐qPCR: *Phenylalanine ammonia‐lyase* (*PAL*), *Lipoxygenase 2.3* (*LOX*); *1‐aminocyclopropane‐1‐carboxylate oxidase 1‐like* (*ACO1*); *Pathogenesis‐related protein 1‐like* (*PR1*); *Chitinase 1* (*CHI1*); *Thaumatin‐like protein 1* (*THA1*). The *Eukaryotic translation initiation factor* (*eTIF*) was used as the endogenous reference gene for normalization (Supplementary Fig. S1). Water treatment (H_2_O) was used as the calibrator for relative gene expression calculations (2^−ΔΔCt^ method). Mean fold change values ± SEM are shown (*n* = 3). Significant differences are marked by * according to one‐way ANOVA and Dunnett's multiple comparisons test (*P* < 0.05).

The expression of defense‐related genes was analyzed by RT‐qPCR in seedlings grown in the presence of CP. As visible in Fig. 4, seedling growth inhibition caused by CP in *L. multiflorum* was associated, at the fourth day of growth, with the overexpression of the ethylene‐biosynthesis gene *ACO1*, an enzyme converting 1‐aminocyclopropane‐1‐carboxylic acid (ACC) into ethylene,^44^ and *chitinase 1* (*CHI1*), a PR protein encoding a fungal chitin‐degrading enzyme.^45^, ^46^ In contrast, no significant variations were detected concerning the genes *Phenylalanine ammonia‐lyase* (*PAL*), encoding the first enzyme of the phenylpropanoid pathway,^47^ *Lipoxygenase 2.3* (*LOX*), encoding the enzyme catalyzing the first step of lipid oxidation for the biosynthesis of jasmonate (JA),^48^ *Pathogenesis‐related protein 1‐like* (*PR1*), a marker of SA‐dependent defenses,^49^ and *Thaumatin‐like protein 1* (*THA1*), encoding a PR protein reported to have antimicrobial activity in various plant species.^50^

### 3.5 Inhibitory activity of CP on different weed species

**Figure 5:**
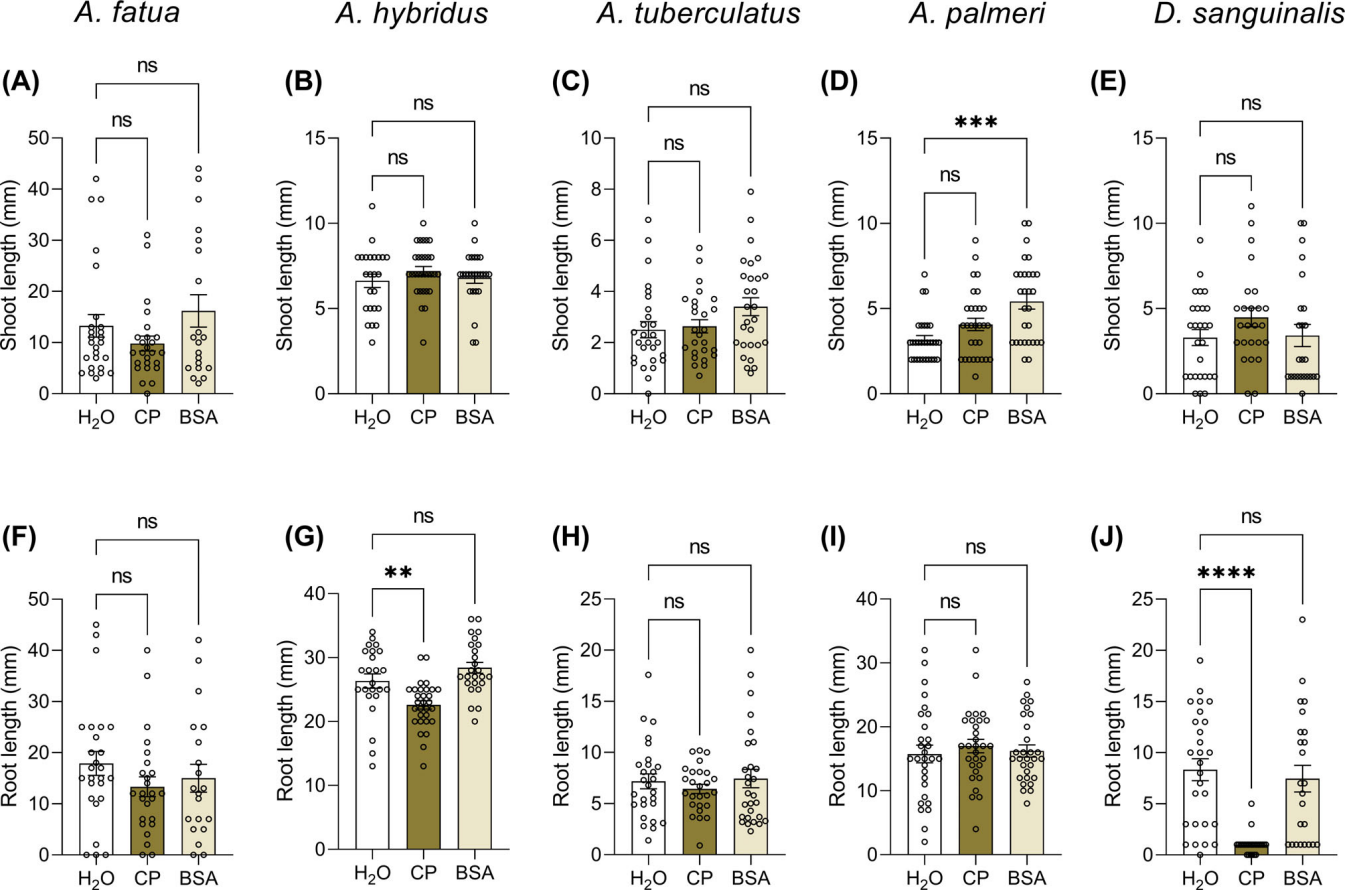
Inhibitory effect of cerato‐platanin (CP) on different weed species. *Avena fatua* (A,F), *Amaranthus hybridus* (B,G), *A. tuberculatus* (C,H), *A. palmeri* (D,I), and *Digitaria sanguinalis* (E,J) seeds were vernalized at 4 °C in the presence of 150 μM CP for different periods of time, in accordance with the requirements of each weed species (Table 2). Shoot and primary root measurements were performed on germinated seeds after 4 days (*A. tuberculatus*, *n* = 26–28), 5 days (*A. fatua n* = 20–26; *A. hybridus*, *n* = 24–30; *A. palmeri*, *n* = 29–30) or 6 days of growth (*D. sanguinalis*, *n* = 24–27). Sterile water (H_2_O) and Bovine Serum Albumin (BSA) were used as controls. Statistical significance was analyzed by non‐parametric Kruskal–Wallis test followed by Dunn's multiple comparisons test at *P* < 0.05. Graphs show average values ± SEM. Significant differences are indicated with **: *P* < 0.01, ***: *P* < 0.001, or ****: *P* < 0.0001: ns, not significant. The experiments were repeated with similar results.

The promising results obtained on *L. multiflorum* prompted us to investigate the sensitivity of other important arable weeds to the eliciting activity of CP in terms of seedling growth inhibition. As shown in Fig. 5, the treatment with CP did not significantly affect *A. fatua*, *A. tuberculatus* and *A. palmeri* seedling growth (*A. palmeri* seeds treated with BSA showed increased shoot length, irrelevant for this study, Fig. 5(D)), whereas CP strongly inhibited *D. sanguinalis* root growth (>85%) (Supplementary Fig. S2) and moderately inhibited *A. hybridus* root growth (approximately 15%). Therefore, CP showed a partially selective inhibitory activity on weeds and, differently from what was observed on *L. multiflorum*, the inhibition on *D. sanguinalis* and *A. hybridus* only occurred at the root level.

### 3.6 *Lolium* populations resistant to ACCase inhibitors are susceptible to CP

**Figure 6:**
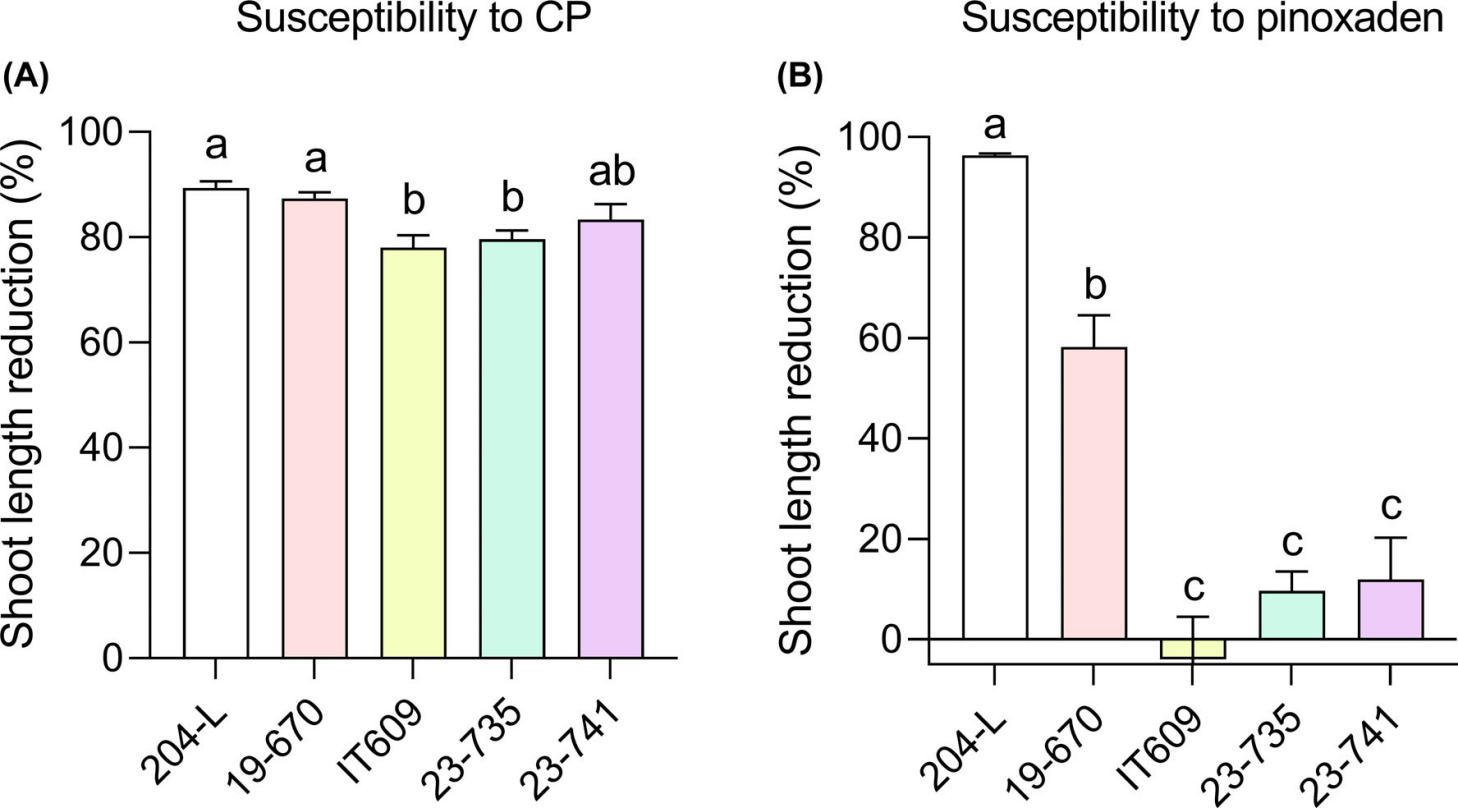
Efficacy of cerato‐platanin (CP) on *Lolium* spp. populations resistant to pinoxaden. The pinoxaden‐susceptible population 204‐L of *L. multiflorum* (control) and four field populations of *Lolium* spp. (19–670, IT609, 23–735, 23–741) showing different frequency of plants resistant to pinoxaden were treated with 500 μL of 150 μM CP (A), by using the Petri dish assay described in Materials and Methods. CP was added after vernalization and shoot length measurements were performed after 7 days of growth. The resistance of the selected populations to the ACCase inhibitor pinoxaden was confirmed by treating the seeds with 500 μL of Axial® Pronto 60 (Syngenta Italia), appropriately diluted (B). The population IT609 is also resistant to acetolactate synthase (ALS) inhibitors.^37^ Values are expressed as mean reduction percentage as compared to the corresponding water‐treated control ± SEM. Letters indicate statistically significant differences at *P* < 0.05 (Kruskal–Wallis test followed by Dunn's multiple comparisons test, *n* = 26–30). The experiment was repeated with similar results.

To demonstrate that CP, by exploiting a different mode of action, can be a promising alternative to reduce growth of herbicide resistant weeds, we treated four *Lolium* spp. populations resistant to the ACCase inhibitor pinoxaden. The population 204‐L of *L. multiflorum* was used as a susceptible control. As visible in Fig. 6(A),(B), CP and pinoxaden strongly inhibited shoot development in the population 204‐L. However, while the populations 19–670, IT609, 23–735 and 23–745 confirmed the presence of different levels of resistance to pinoxaden (Fig. 6(B)), they were all highly susceptible to CP (shoot length reduction always ≥78%) (Fig. 6(A)).

### 3.7 Cerato‐platanin does not affect wheat germination and seedling growth

**Figure 7:**
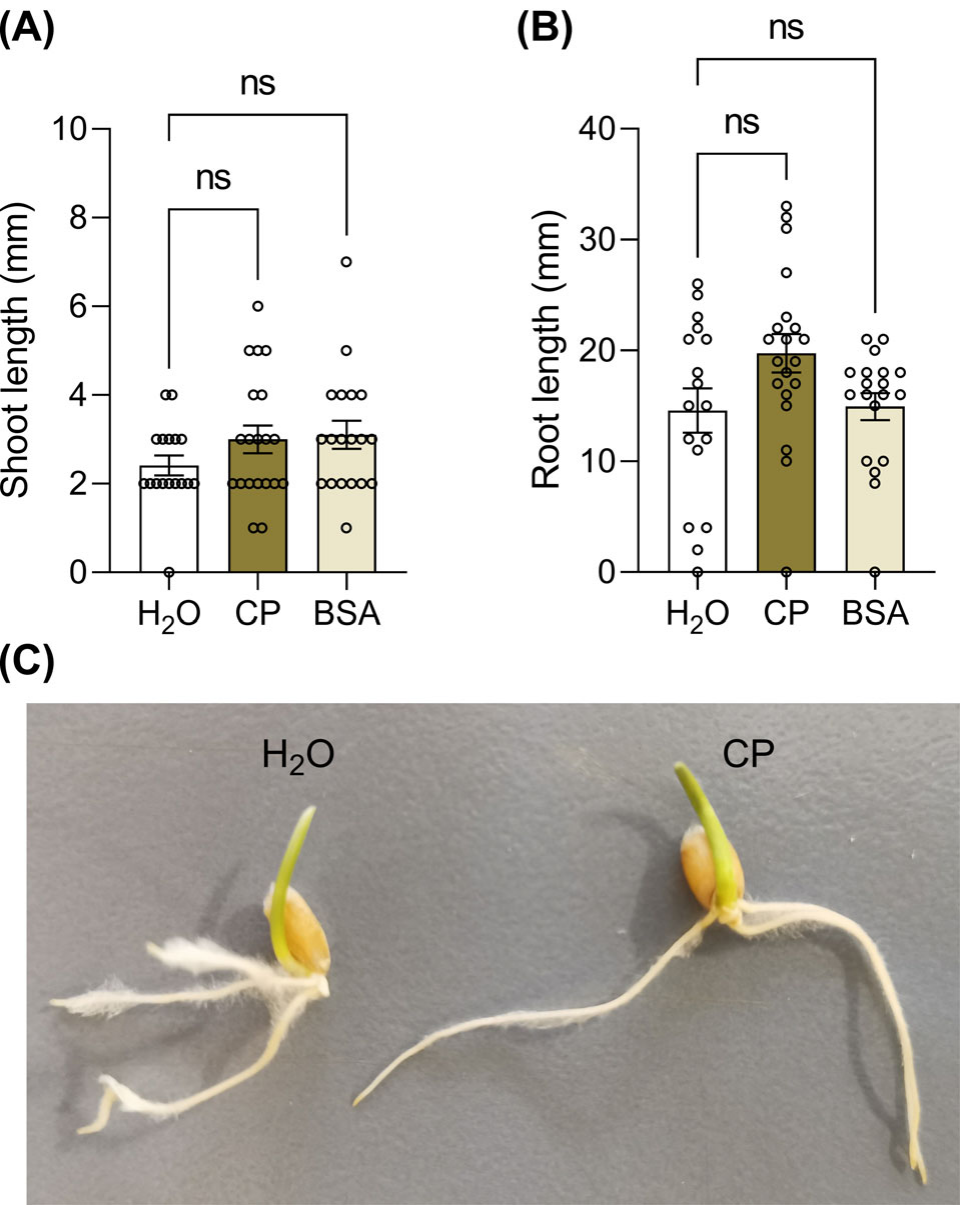
Off‐target activity of cerato‐platanin (CP) on wheat. *Triticum aestivum* seeds were vernalized 4 days at 4 °C in the presence of 150 μM CP, sterile water, or 150 μM Bovine Serum Albumin (BSA), and germinated 4 days before shoot (A) and primary root (B) length measurements. Non‐germinated seeds were excluded from measurements. Graphs show average values ± SEM. Statistical significance was analyzed by non‐parametric Kruskal–Wallis test followed by Dunn's multiple comparisons test at *P* < 0.05 (*n* = 17–20). ns, not significant. The experiment was repeated with similar results. A representative picture of a wheat seedling germinated in the presence of water or CP is shown (C).

Wheat was selected as the species to be assessed for off‐target inhibitory effects by CP, since *L. multiflorum* seriously affect wheat production.^51^ As shown in Fig. 7, the treatment with CP did not significantly affect wheat seedling growth after 4 days of growth as compared to water treatment, neither did it reduce germination rate (100% for CP). Unlike *L. multiflorum*, CP was not detectable in wheat seedlings after 4 days of growth (Supplementary Fig. S3).

## 4 DISCUSSION

Natural microbial compounds may affect cellular functions different from those of commercial herbicides and provide new modes of action to fight resistant weeds. In this regard, this study shows the herbicidal potential of a fungal protein targeting the immune system of plants.

Over the last decades, huge progress has been made on the understanding of how plants and pathogenic microbes interact at the molecular level.^16^ Nevertheless, arable weeds have received little attention in that kind of study and the available scientific literature is scarce in information. In particular, the sensitivity of arable weeds to PAMPs is mostly unknown.^17^, ^18^, ^19^, ^20^, ^27^

In this work, the PAMP protein cerato‐platanin (CP), a natural product of the plant pathogenic fungus *C. platani*,^33^ was applied to agronomically important weed species belonging to Poaceae (*L. multiflorum, A. fatua, D. sanguinalis*) and Amaranthaceae (*A. hybridus, A. palmeri, A. tuberculatus*), with the aim of taking advantage of the growth‐defense tradeoff, a natural mechanism intimately connected to the plant immune reaction.^22^, ^23^

In the literature, CP has been demonstrated to induce resistance against pathogens and PTI‐related cellular responses, such as reactive oxygen species and nitric oxide production, transcription of defense‐related genes, phytoalexin synthesis, stomatal closure and cell death in leaves from distantly related plant species, such as *A. thaliana* (Brassicaceae)^31^ and *P. acerifolia* (Platanaceae),^30^ suggesting an apparent widespread perception among plants. Treatment with CP has also been found to reduce the amount of primary metabolism enzymes (e.g., ribulose‐1,5‐bisphosphate carboxylase/oxygenase‐RuBisCO) and decrease photosynthetic activity in *A. thaliana*,^41^ implying the occurrence of growth penalties following treatment with the protein. In this study, the eliciting activity of CP was exploited to hamper weed growth.

Our data show that CP can reduce seedling growth in *L. multiflorum* while it does not negatively affect the germination rate. The inhibition observed on *L. multiflorum* was dose‐dependent, specific to the treatment with CP and resulting from the absorption of the protein, which caused the activation of pathogen defense, in accordance with plant responses to PAMP elicitors.^17^ CP‐specific inhibition was observed with concentrations ≥75 μM, as supported by the dose–response assay, but 150 μM turned out to be the optimal dose to inhibit both shoot and root development in ryegrass. With our study we are also able to highlight the application potential of this inhibition, because CP was effective on *Lolium* spp. populations resistant to the ACCase inhibitor pinoxaden. Among the populations tested, we included the *Lolium* spp. population IT609, whose characterization in a previous study has allowed revealing the presence of target‐site and metabolism‐based resistance mechanisms to different ACCase and ALS inhibitors.^37^

Our results also revealed different sensitivity and response among plant species to CP, a feature never reported before for this protein but known for other PAMPs,^52^ for instance flagellin or its active epitopes.^28^, ^53^, ^54^ Weed response after treatment with biomolecules or plant extracts has frequently been reported to depend on the weed species and doses applied.^55^ Different characteristics of the seeds, such as size and permeability,^56^ or the environmental factors present in the field^57^ may influence the herbicidal effect. However, in this context, we hypothesize that the major determinant governing sensitivity of the tested species to CP is the presence/absence of a CP receptor, or more likely its different binding affinity for the protein. Indeed, CP‐family proteins are perceived by plants as PAMP by a still uncharacterized pattern‐recognition receptor (PRR) that starts, after binding, the defense signaling pathway and leads to PTI.^19^, ^32^, ^35^ It is known from the literature that different plants may sense different epitopes of a PAMP, and polymorphisms in these regions may affect immune recognition.^54^, ^58^, ^59^ Therefore, species insensitive to CP could however sense other CP‐family proteins.^32^

In *A. thaliana*, the defense response triggered by CP was previously reported to be associated to the overexpression of ethylene‐ and salicylic acid‐dependent genes, downregulation of jasmonic acid‐dependent genes, and overexpression of genes encoding some pathogenesis‐related (PR) proteins (e.g., chitinases and PR1, but not thaumatin) in treated leaves during the first 24 h.^31^ The gene expression analyses performed in *L. multiflorum* seedlings at the fourth day of growth showed a partial overlap with the gene modulation previously reported in *A. thaliana*, with the overexpression of an ethylene‐biosynthesis gene (*ACO1*), the overexpression of the PR gene *chitinase 1* (*CHI1*) and lack of modulation for the PR gene *Thaumatin‐like protein 1* (*THA1*). The relationship between ethylene perception and chitinase production in plants, which do not contain chitin in their cell wall, has been known for a long time as defense mechanism against microbial invaders.^60^ Therefore, the induction of both *ACO1* and *CHI1* genes in *L. multiflorum* suggests the activation of antimicrobial defense mechanisms by CP responsible for the growth penalties detectable on seedlings. In contrast, salicylic acid‐ and jasmonic acid‐dependent genes (*PAL*, *PR1* and *LOX*) were unaffected in our experimental conditions, likely because of the different treatment times (hours *vs*. days) and mode of application of CP (leaves *vs*. seeds) between the two studies.

To conclude, treatment of *Triticum aestivum* with CP did not result in seedling growth inhibition and traces of the protein could not be found. This result suggests that CP was likely totally absorbed and degraded on the fourth day of growth, time of sampling. Our hypothesis is that CP, ineffective on this species because not sensed, can be rapidly absorbed and degraded by proteases that normally hydrolyze seed storage proteins to provide amino acids essential for embryo growth and development.^61^ The insensitivity of wheat to CP is relevant from the weed management perspective, because it opens the possibility to provide the crop with an important growth advantage on its weeds such as ryegrass, in a targeted manner, thereby making it more competitive. Crop competitiveness for weed suppression is not a new concept, but the need for sustainable alternatives to manage resistant weeds has given a new boost to this research area.^62^

The biopesticide sector is investing resources in startup companies developing peptides for crop protection,^11^ although their use as herbicides is still in its infancy.^63^, ^64^ Natural proteins and peptides would be ideal candidates to replace/reduce harmful active ingredients in novel (bio)herbicides, since they are non‐volatile and can be easily degraded into amino acids when in soil. This work highlights the potential of this kind of molecule to provide sustainable solutions for the lack of new modes of action and the problem of weed resistance.

## 5 CONCLUSION

Plant immune stimulators are classically studied, and in some cases marketed, as resistance inducers against pests and diseases. This study supports the potential use of natural immune stimulators from microbes, such as CP and very likely other PAMP proteins and peptides, against weeds. We propose that herbicide resistant weeds, which require innovative modes of action to be controlled, can particularly benefit from the exploitation of this class of plant immune activators.

## CONFLICT OF INTEREST

The authors declare that they have no competing interests.

## Supporting information


**Table S1.** Genes analyzed by RT‐qPCR in *Lolium multiflorum* and primer sequences.
**Table S2.** Germination rate (%) of *Lolium multiflorum* 204‐L in the presence of increasing concentrations of CP or bovine serum albumin (BSA).
**Figure S1.** Transcriptional stability of three candidate endogenous reference genes.
**Figure S2.** Representative picture of the effect caused by CP on *D. sanguinalis*.
**Figure S3.** Western blot anti‐CP of lysates of *T. aestivum* seedlings.

**Dataset Figure S1.** *Lolium multiflorum* shoot/root length (mm) measurements.

**Dataset Figure S2.** *Lolium multiflorum* shoot/root length (mm) measurements (dose‐response assay).

**Dataset Figure S4.** Ct values from RT‐qPCR analyses.

**Dataset Figure S5.** Shoot/root length (mm) measurements on different weed species.

**Dataset Figure S6.** Shoot length (mm) measurements in herbicide resistant *Lolium* spp. populations.

**Dataset Figure S7.** *Triticum aestivum* shoot/root length measurements (mm).

## Data Availability

The data that supports the findings of this study are available in the supplementary material of this article.
