# Supplementary material for: Growth inhibition of arable weeds by cerato‐platanin, a plant immune defense activator of fungal origin
Source: Pest Manag Sci. 2025 Jun 24;81(10):6255–64. doi: 10.1002/ps.8963 (PMC12441781; doi:10.1002/ps.8963)

**SUPPORTING INFORMATION**

**Table S1.** Genes analyzed by RT-qPCR in *Lolium multiflorum* and primer sequences.

| **Gene** | **Forward primer (5’-3’)** | **Reverse primer (5’-3’)** |
| --- | --- | --- |
| Reference genes |  |  |
| *Elongation factor 1-alpha (EF1A)* | GACAAGCCCCTGCGTCTTC | CACAGTTCCAATGCCACCAA |
| *Eukaryotic translation initiation factor (eTIF)* | GGAGCACTGATTGGCAAGAGT | AGGCTGGAAGGGCGAGAT |
| *Actin 11 (ACT11)* | TTGTGTGCGACAATGGAACTG | CGCATCATCCCCAGCAA |
| Defense-related genes |  |  |
| *Lipoxygenase 2.3* *(LOX)* | CCTCCCTTCGCAGATGCA | GCGAGTGGGAGGAAAAAATG |
| *Phenylalanine ammonia-lyase (PAL)* | AGCCTGGACTACGGGTTCAA | ACCGGGTTTGCGAGGTACT |
| *1-aminocyclopropane-1-carboxylate oxidase 1-like (ACO1)* | GCGCTTGTTGCGAAGGA | CGCACGTACAGCTTCATGTAGTC |
| *Pathogenesis-related protein 1-like (PR1)* | TCGCGTGGTCTGCAACAA | CGTGGGTCGTAGCTGCAGAT |
| *Chitinase 1 (CHI1)* | CATATCCCAGTCGCTCTTCGA | GAAGCCCGAGAAGGAGTTAGC |
| *Thaumatin-like protein 1 (THA1)* | GACGGCACATCCACCTTCAC | ACTGTGGGCAGAAGGTAATGGT |

**Table S2.** Germination rate (%) of *Lolium multiflorum* 204-L in the presence of increasing concentrations of CP or Bovine Serum Albumin (BSA). Rates are shown for single replicates. Shoot and root length measurements are reported in Figure 2 where replicates are merged.

| **Treatment (replicate)** | **Protein concentration (µM)** | | | | | |
| --- | --- | --- | --- | --- | --- | --- |
|  | **0** | **37.5** | **75** | **150** | **300** | **600** |
| Water (1) | 96.7 |  | | | | |
| Water (2) | 100.0 |  |  |  |  |  |
| Water (3) | 100.0 |  |  |  |  |  |
| CP (1) |  | 96.7 | 100.0 | 96.7 | 100.0 | 100.0 |
| CP (2) |  | 100.0 | 80.0 | 100.0 | 96.7 | 100.0 |
| CP (3) |  | 96.7 | 100.0 | 100.0 | 96.7 | 96.7 |
| BSA (1) |  | 96.7 | 100.0 | 100.0 | 93.3 | 100.0 |
| BSA (2) |  | 100.0 | 93.3 | 100.0 | 96.7 | 86.7 |
| BSA (3) |  | 96.7 | 100.0 | 100.0 | 100.0 | 96.7 |

**Figure S1.** Transcriptional stability of three candidate endogenous reference genes (*Elongation factor 1-alpha, EF1A; Eukaryotic translation initiation factor, eTIF;* and *Actin 11, ACT11)* across all different cDNA samples from each experimental condition (Water, BSA and CP with three biological replicates). **A**, threshold cycles (Ct) from two technical replicates, average Ct value and standard deviation (SD) of each gene are reported. **B**, Ct values are shown in graph.

**A**

| **Seed treatment** | **Sample Nr.** | ***EF1A***  **Ct values** | ***eTIF***  **Ct values** | ***ACT11***  **Ct values** |
| --- | --- | --- | --- | --- |
| Water | H2O-1 | 18,86291 | 25,32789 | 22,02003 |
| Water | H2O-2 | 19,02794 | 25,9198 | 21,61641 |
| Water | H2O-3 | 18,47059 | 25,1992 | 22,21008 |
| BSA, 150µM | BSA-1 | 18,55546 | 25,4119 | 21,98851 |
| BSA, 150µM | BSA-2 | 18,46681 | 25,12756 | 21,66883 |
| BSA, 150µM | BSA-3 | 18,41901 | 25,02469 | 21,6293 |
| CP, 150µM | CP-1 | 20,31518 | 26,36586 | 23,12207 |
| CP, 150µM | CP-2 | 18,59102 | 24,68536 | 21,89131 |
| CP, 150µM | CP-3 | 18,97094 | 25,43988 | 22,31855 |
|  |  |  |  |  |
|  | **Average Ct value** | 18,85 | 25,39 | 22,05 |
|  | **SD** | 0,59 | 0,50 | 0,47 |

**B**

**Figure S2.** Representative picture of the root inhibition effect caused by 150 µM CP on *Digitaria sanguinalis*, as compared to 150 µM BSA and water, after 6 days of growth.


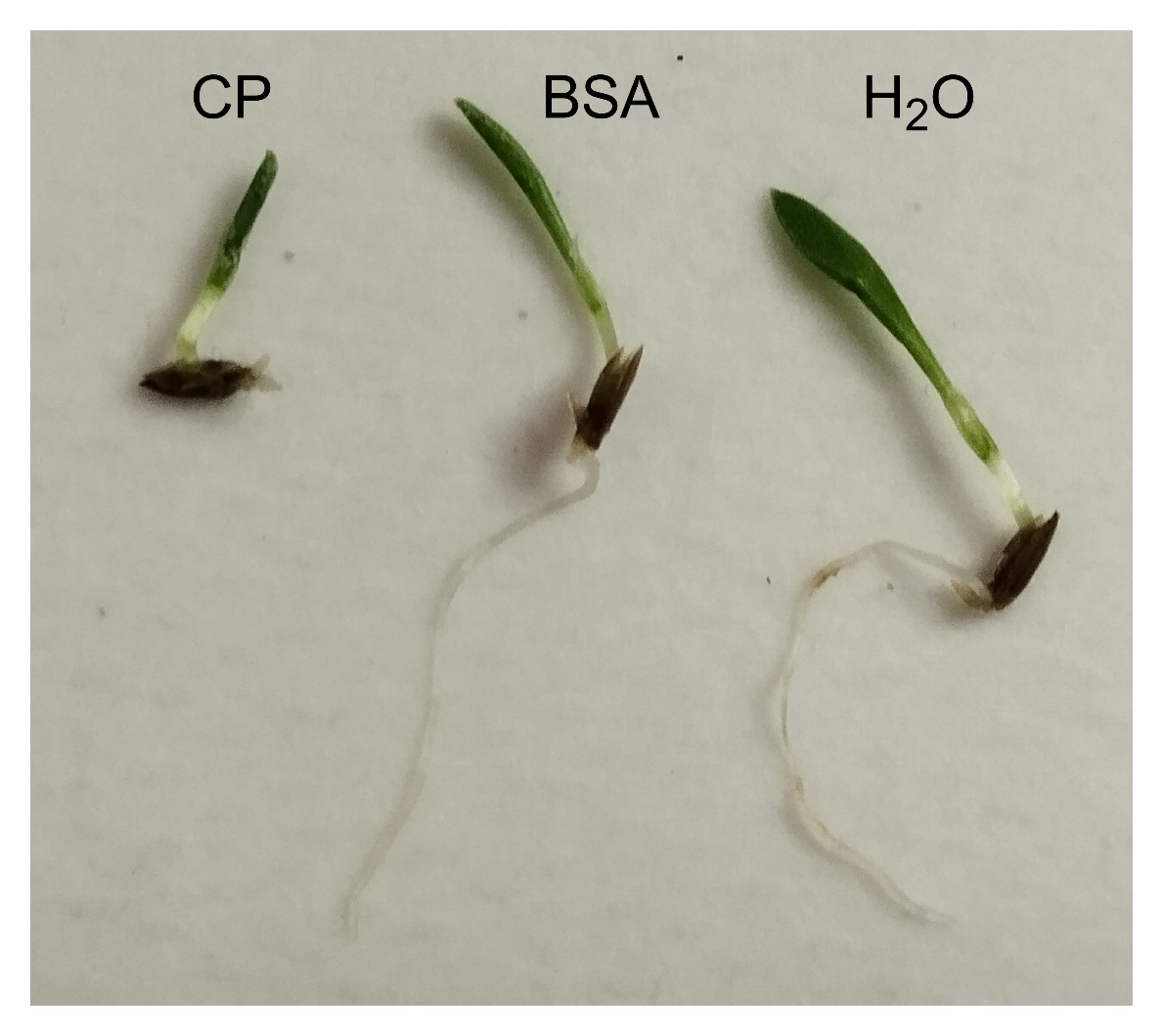


**Figure S3.** Western blot anti-CP of lysates of *T. aestivum* seedlings vernalized (4 days) and grown (4 days) in the presence of 150 µM CP, or water as a control. Samples were collected without rinsing from three different Petri dishes containing CP or water. A replicate experiment with *Lolium multiflorum* was performed and analyzed in parallel for comparison. Pure CP (3.2 µg) was loaded as a positive control. Std, Precision Plus Protein Kaleidoscope™ Prestained Protein Standards #1610375. A rectangle was drawn to show where the band corresponding to CP was expected.


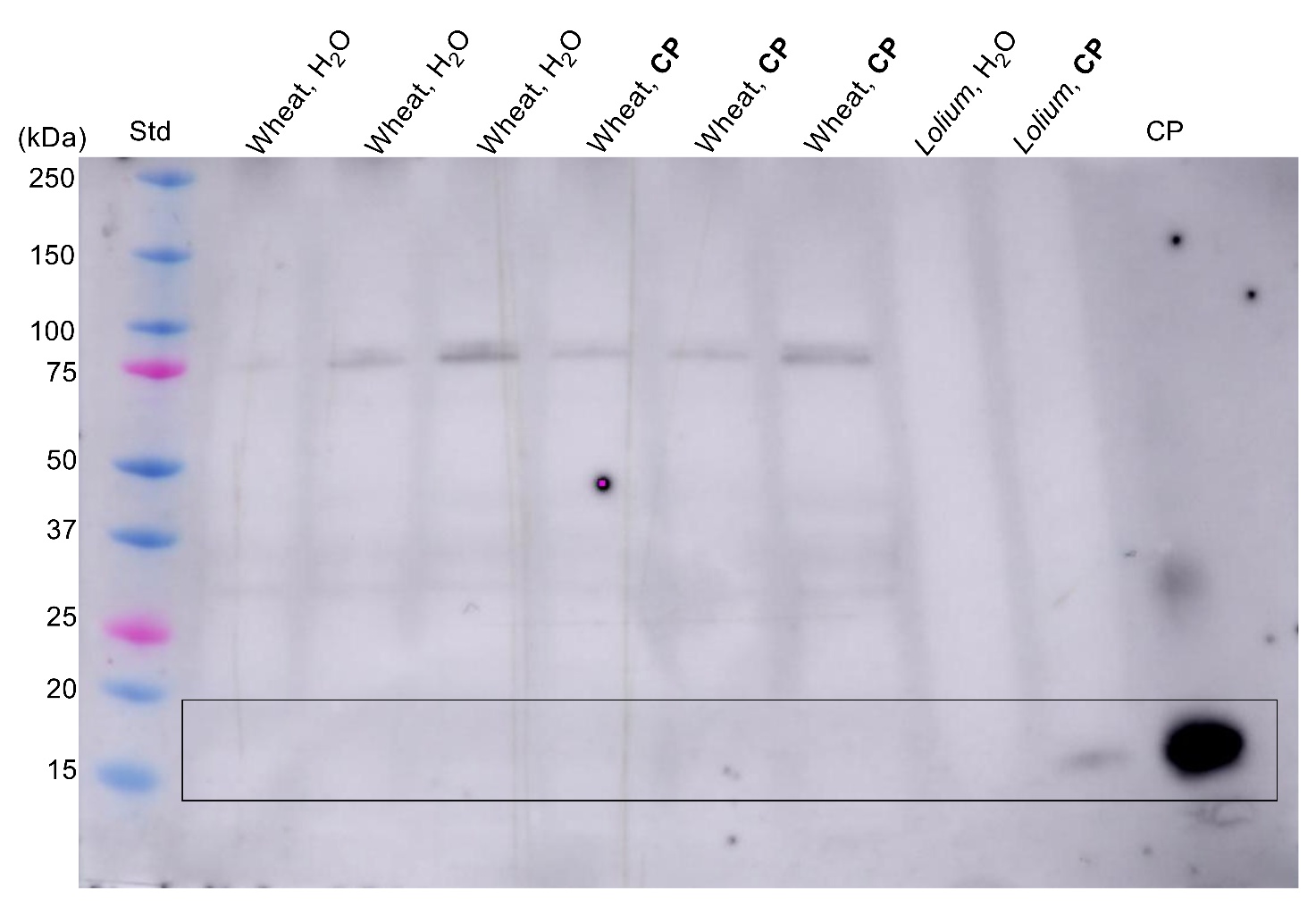

Supplement: Supplementary file 1 — Table S1. Genes analyzed by RT‐qPCR in Lolium multiflorum and primer sequences. Table S2. Germination rate (%) of Lolium multiflorum 204‐L in the presence of increasing concentrations of CP or bovine serum albumin (BSA). Figure S1. Transcriptional stability of three candidate endogenous reference genes. Figure S2. Representative picture of the effect caused by CP on D. sanguinalis. Figure S3. Western blot anti‐CP of lysates of T. aestivum seedlings. [file PS-81-6255-s004.docx]
